# Pathologist performed fine needle aspirations & implementation of JCAHO Universal Protocol and "Time out"

**DOI:** 10.1186/1742-6413-4-19

**Published:** 2007-09-20

**Authors:** Momin T Siddiqui

**Affiliations:** 1 Department of Pathology and Laboratory Medicine, Emory University Hospital, Atlanta, Georgia, USA

## Abstract

The adherence to the principles of the Universal Protocol for preventing wrong site, wrong procedure and wrong person surgical or invasive procedures is a requirement for all Joint Commission accredited organizations. Fine needle aspirations are considered invasive procedures, and cytopathologists performing this procedure need to be cognizant and compliant with the requirements of this Joint Commission on Accreditation of Healthcare Organizations (JCAHO) Protocol. This article gives background perspective on the development of the Universal Protocol. It also elaborates the JCAHO National Patients Safety Goals regarding the performance of fine needle aspirations. The compliance with the Universal Protocol for performance of fine needle aspirations is now mandated for all cytopathologists who perform fine needle aspirations and this present paper provides a guideline for fulfilling the requirements of the Universal Protocol for practicing cytopathologists.

## Background

The subject of wrong site surgery and factors leading to it, have been a topic of discussion in medical and legal publications as well as mainstream press [1]. This issue has become prominent because of profound patient care, social and medico legal consequences. A report by the institute of Medicine estimated that 44,000 to 98,000 individuals in the United States die each year of preventable medical errors, which have an estimated cost of approximately 9 billion dollars to United States taxpayers [2,3]. Fine needle aspirations are considered invasive procedures which depending on the diagnosis rendered, lead to definitive surgical procedures. This can profoundly impact the practice of a fine needle aspiration service in clinical setting, and hence JCAHO requiring implementation of the Universal Protocol and "**time out**" for these procedures. The "time out" or immediate preprocedural process is the implementation and expectation of completion, for JCAHO mandated requirements.

### Development of the Universal Protocol

The Joint Commission on Accreditation of Healthcare Organizations (JCAHO), hosted a Wrong Site Surgery Summit on May 9, 2003 [4,5]. The goal of this summit was to obtain a consensus on the adoption of a "Universal Protocol" for preventing wrong site, wrong procedure and wrong person surgical or invasive procedures, which includes fine needle aspirations. The Summit was hosted by the JCAHO in collaboration with various organizations including the American Medical Association and American College of Physicians [4,5]. The participants, which included 30 other professional groups agreed that a Universal Protocol would help prevent the occurrence of wrong site, wrong procedure and wrong person surgical or invasive procedures. They also agreed that the Protocol should be specific and eliminate confusion about site marking and facilitate communication among patient care members.

A broad consensus on the draft of the Universal Protocol was sought, subsequent to which, JCAHO approved the Universal Protocol for Preventing Wrong Site, Wrong Procedure and Wrong Person Surgery™ in July 2003 [4,5]. This Universal Protocol was thus created to address the continuing occurrence of medical errors in JCAHO accredited organizations and became effective July 1, 2004 for all accredited hospitals, ambulatory care and office-based surgery facilities [4,5]. The Universal Protocol is applicable to all operative and other invasive procedures, including pathologist performed fine needle aspirations. The principal components of the Universal Protocol include 1) The pre-operative verification process; 2) marking of the operative site; 3) taking a "time out" immediately before starting the procedure; and 4) adaptation of the requirements to non-operating room settings, including clinic and bedside procedures. These principal components of the Universal Protocol are further elaborated in Tables 1, 2, 3, 4, [4,5].

**Table 1:** Pre-operative verification process (modified from references 4 and 5)

	Verification of the correct person, procedure, and site should occur (as applicable):
1.	At the time the surgery/procedure is scheduled.
2.	At the time of admission or entry into the facility.
3.	Anytime the responsibility for care of the patient is transferred to another caregiver.
4.	With the patient involved, awake and aware, if possible.
5.	Before the patient leaves the preoperative area or enters the procedure/surgical room.
	A preoperative verification checklist may be helpful to ensure availability and review of the following, prior to the start of the procedure:
1.	Relevant documentation (e.g., History and physical examination, consent).
2.	Relevant images properly labeled and displayed.
3.	Any required implants and special equipment.

**Table 2:** Marking the operative site (modified from references 4 and 5)

1.	Make the mark at or near the FNA procedure site. Do not mark any non-operative site(s) unless necessary for some other aspect of care.
2.	The mark must be unambiguous (e.g., use initials or "YES" or a line representing the proposed incision; consider that "X" may be ambiguous)
3.	The mark must be positioned to be visible after the patient is prepped and draped.
4.	The mark must be made using a marker that is sufficiently permanent to remain visible after completion of the skin prep. Adhesive site markers should not be used as the sole means of marking the site.
5.	The method of marking and type of mark should be consistent throughout the organization and be used by all pathologists.
6.	At a minimum, mark all cases involving laterality, multiple structures (fingers, toes, lesions), or multiple levels (spine).
7.	The person performing the procedure should do the site marking.
8.	Marking must take place with the patient involved, awake and aware.
9.	Final verification of the site mark must take place during the "time out."
10.	A defined procedure must be in place for patients who refuse site marking.

**Table 3:** "Time out" immediately before starting the procedure (modified from references 4 and 5)

	Must be conducted in the location where the procedure will be done, just before starting the procedure. It must involve the entire operative team, use active communication, be briefly documented, such as in a checklist (the organization should determine the type and amount of documentation) and must, at the least, include:
1.	Correct patient identity.
2.	Correct side and site.
3.	Agreement on the procedure to be done.
4.	Correct patient position.
5.	Availability of correct implants and any special equipment or special requirements.
	The organization should have processes and systems in place for reconciling differences in staff responses during the "time out."

**Table 4:** Procedures for non-operating room settings including bedside procedures (modified from references 4 and 5)

1.	Site marking must be done for any procedure that involves laterality, multiple structures or levels (even if the procedure takes place outside of a surgical operating room).
2.	Verification, site marking, and "time out" procedures should be as consistent as possible throughout the organization, including the operating rooms and other locations where invasive procedures are done.
3.	Exception: Cases in which the individual doing the procedure is in continuous attendance with the patient from the time of decision to do the procedure and consent from the patient through to the conduct of the procedure may be exempted from the site marking requirement. The requirement for a "time out" final verification still applies.

### JCAHO National Patient Safety Goals

The 2007 JCAHO National Patient Safety Goals address the specific application of the Universal Protocol to invasive procedures performed by the clinical laboratory professionals, which includes pathologists performing fine needle aspirations [6]. The goal that applies to pathologists performing fine needle aspirations is as follows:

#### Goal 1: Improve the accuracy of patient identification

The rationale for this goal is to prevent the occurrence of wrong patient identification. Hence, the intent for this goal is two fold: first to reliably identify the individual as the person for whom the service or treatment is intended and secondly, to match the service provided to that patient. This goal has two main requirements, and the failure to comply with these requirements may trigger loss of JCAHO accreditation status, for JCAHO accredited institutions.

#### Requirement 1A

This requirement states that at least two patient identifiers (neither to be a room number) be used, whenever collecting laboratory samples, which also include pathologist performed, fine needle aspirations. To implement this requirement for fine needle aspirations, acceptable identifiers may be the patient's name, an assigned identification number, telephone number, photograph, or other person-specific identifiers. A bar code on a wrist band that includes two or more person-specific identifiers will comply with this requirement. This requirement covers the pre-procedural verification process, and should be performed before the fine needle aspiration procedure is performed.

#### Requirement 1B

This requirement states that prior to the start of any invasive procedure, conduct a final verification process to confirm the correct patient, procedure, site and availability of appropriate documents. This verification process uses active, and not passive, communication techniques. The patient's identity has to be re-established if the pathologist leaves the patient's location, prior to initiating the procedure. Marking the site is also required unless the practitioner is in continuous attendance from the time of the decision to do the procedure and patient consent to the initiation of the procedure [2]. The mark must be made using initials or "YES", since "X" is considered ambiguous. The mark must be made using a marker that is sufficiently permanent to remain visible after completion of the skin prep, with alcohol. The implementation and expectation of completion, for this requirement, is called "**time out**".

The "time out" is the immediate preprocedural process, and must occur in the location where the procedure is to be done (for example, when the patient is in the fine needle aspiration clinic). The "time out" should involve the entire procedural team which, at a minimum, includes the pathologist doing the procedure, the anesthesia provider (if any), and the circulating nurse, resident, or other assistant. In addition, there should be no barrier to anyone speaking up if there is a concern about a possible error. "Active" communication, in this context, means an affirmation, orally or by some action that the patient, procedure and site are correct.

## Discussion

An important component of fostering a culture of patient safety in a health care setting is having all health care providers adopt common procedures and safeguards to decrease the risk of harm to patients [7]. This is the goal of the Universal Protocol that was implemented nationwide in 2004 to help prevent medical injuries and deaths caused by wrong-site, wrong-procedure or wrong-identity of a patient during surgery or other invasive procedures, including pathologist performed fine needle aspirations [8,9].

Like most hospitals, the Emory University Hospital has implemented this Protocol and has put into place a policy and process for clinicians, including pathologists, to follow. The first step is to verify the patient's identity using at least two patient identifiers, which can be the patient's name, birth date or medical record number. Then the intended procedure also is confirmed by checking the procedure consent form, the history or progress notes, nursing assessment and, if applicable, the procedure/surgical operating room schedule (Table 1) [4,5].

The second step is that the procedure site must be properly marked to identify the area of the patient's body that will undergo the procedure before an incision is made (Table 2) [4,5]. For example, if a patient is having a fine needle aspiration of the left breast, the pathologist would write "YES" on the patient's left breast area to distinguish the site from the right breast. The patient is also an integral part of this safety practice and participates by verifying his or her identity as well as confirming correct procedure, correct side and site.

Before the procedure starts, a third step or "**time out**" is initiated by the physician conducting the procedure to give all members of the clinical team an opportunity to perform a final verification of the correct patient, correct procedure, correct side, correct site, correct patient position and availability of any special equipment or requirements that are needed for the fine needle aspiration procedure (Table 3) [4,5].

JCAHO requires that pathologist performed fine needle aspiration procedures adhere to the rules of the Universal Protocol including the implementation of "time out" for these procedures. The "time out" procedure also needs to be documented for JCAHO inspectors to review. This can be done either by documenting "time out" as a note in the patient's medical file, which is retained as a hard copy in the patient's medical records. Alternatively, "time out" can be also be documented by incorporating it as part of the fine need aspiration report. A text reading out as **"The patient is identified using two identifiers. The site of the fine needle aspiration procedure is also verified with the patient. The lesion is palpated, correctly identified, and marked with ink during time out completion"**, incorporated in the Fine needle aspiration report will conform completion of "time out" for the procedure.

At our institution, the Universal Protocol has been implemented for all pathologist performed fine needle aspirations. This has been in effect since January 01, 2005. In the years 2005 and 2006, a total of 1,779 and 2,079 fine needle aspirations, respectively, were performed by pathologists. In reviewing this data, no errors were detected based on wrong person or wrong site. In summary, at our institution, the implementation of the Universal Protocol has been successful, since conforming to it necessitates that appropriate time be allocated for its compliance and hence prevent any error in patient identity or site identification, for patients about to undergo a fine needle aspiration.
